# The Role of Interleukin-17A and NLRP3 Inflammasome in the Pathogenesis of Graves’ Ophthalmopathy

**DOI:** 10.3390/life13041007

**Published:** 2023-04-13

**Authors:** Chih-Chung Lin, Shu-Lang Liao, Yi-Hsuan Wei

**Affiliations:** 1 Department of Ophthalmology, Taipei City Hospital, Taipei 103212, Taiwan; coolguylin@gmail.com; 2 Department of Ophthalmology, National Taiwan University Hospital, Taipei 100225, Taiwan; liaosl89@ntu.edu.tw

**Keywords:** Graves’ ophthalmopathy, IL-17A, NLRP3 inflammasome, IL-1β

## Abstract

The development of Graves’ ophthalmopathy (GO) is associated with autoimmune dysfunction. Recent studies have indicated that IL-17A, inflammasomes, and related cytokines may be involved in the etiology of GO. We sought to investigate the pathogenic role of IL-17A and NLRP3 inflammasomes in GO. Orbital fat specimens were collected from 30 patients with GO and 30 non-GO controls. Immunohistochemical staining and orbital fibroblast cultures were conducted for both groups. IL-17A was added to the cell cultures, and cytokine expression, signaling pathways, and inflammasome mechanisms were investigated using reverse transcription polymerase chain reaction, enzyme-linked immunosorbent assay, Western blotting, and small interfering RNA (siRNA) methods. Immunohistochemical staining showed higher NLRP3 expression in GO orbital tissue than in non-GO controls. IL-17A upregulated pro-IL-1β mRNA levels and IL-1β protein levels in the GO group. Furthermore, IL-17A was confirmed to enhance caspase-1 and NLRP3 protein expression in orbital fibroblasts, suggesting NLRP3 inflammasome activation. Inhibiting caspase-1 activity could also decrease IL-1β secretion. In siRNA-transfected orbital fibroblasts, significantly decreased NLRP3 expression was observed, and IL-17A-mediated pro-IL-1β mRNA release was also downregulated. Our observations illustrate that IL-17A promotes IL-1β production from orbital fibroblasts via the NLRP3 inflammasome in GO, and cytokines subsequently released may induce more inflammation and autoimmunity.

## 1. Introduction

Graves’ ophthalmopathy (GO), the ocular manifestation of Graves’ disease (GD), usually presents as orbital inflammation, tissue expansion, and fibrosis, and often confers substantial morbidity [1]. The clinical signs of GO may include conjunctival chemosis, periorbital soft tissue swelling, proptosis, restricted eye movement, and visual impairment due to compressive optic neuropathy [2]. The pathogenesis of GO awaits detailed clarification.

T helper 17 (Th17) cells reflect a novel T cell population that mainly produces IL-17A, IL-17F, and IL-22. IL-17 (mainly IL-17A) was reported to induce the production of proinflammatory cytokines, such as tumor necrosis factor-α (TNF-α) and IL-1 in several tissue cell types, such as macrophages and fibroblasts [3]. For example, IL-17 plays an important role in the pathogenesis of rheumatoid arthritis by promoting the proliferation and function of fibroblast-like synoviocytes [3].

Since GO-associated orbital tissue changes also reflect inflammatory processes, the role of IL-17A in the pathogenesis in GO warrants investigation. Recent studies reported an increase in Th17 cells in the orbit of patients with GO [4]. Additionally, IL-17A was observed to promote both inflammation (IL-6 and IL-8 production) and extracellular matrix (ECM) accumulation in orbital fibroblasts [5]. The roles of cytokines and chemokines are complicated and important in the pathogenesis of GO. Furthermore, cytokines and their receptors are potential targets of therapeutic intervention in aggressive and sight-threatening forms of GO [6,7].

Inflammasome activation is a key component of innate immunity [8]. Inflammasomes can be assembled and activated in response to reactive oxygen species (ROS) [9]. They assemble into large complexes, leading to autocatalytic cleavage of pro-caspase-1, resulting in caspase-1 activation. Active caspase-1 can cleave various cytokine precursors, including IL-1β, which is an inciting factor in GO [6]. Elevated intracellular ROS levels in GO orbital fibroblasts were reported, and hypersensitivity to oxidative stress of GO orbital fibroblasts may play a role in the pathogenesis of GO [10,11]. The most studied member of the inflammasomes is NACHT, leucine-rich repeat (LRR), and PYD domain-containing protein 3 (NLRP3).

A recent study of age-related macular degeneration (AMD) reported that IL-17A induces IL-1β secretion from retinal pigment epithelium (RPE) cells via NLRP3 inflammasome activation [12]. This finding successfully confirmed interactions between IL-17A and inflammasomes in the pathogenesis of AMD. Similarly, both IL-17A signaling and inflammasome expression have been linked to the pathogenesis of GO [4]; however, to the best of our knowledge, no current studies have adequately explored the interactions between them. Therefore, we hypothesized that local IL-17A might serve as a trigger for inflammasome signaling and IL-1β production in orbital fibroblasts, resulting in a positive feedback loop.

Here, we sought to investigate the role of IL-17A in the pathogenesis of GO. Moreover, we would like to study not only the role of IL-17A, but also its link to the inflammasome-related phenomenon occurring in the course of Graves’ orbitopathy. Since successful animal models of GO have yet to be established, we mainly used surgical specimens and cultured orbital fibroblasts for the experiments.

## 2. Materials and Methods

### 2.1. Patient Selection

The present study was approved by the Research Ethics Committee of the National Taiwan University Hospital (approval no. 201612045RINB). We collected 30 orbital connective tissue specimens from patients undergoing fatty decompression surgery due to GO, and 30 control orbital fat tissue specimens from patients without GD receiving eye bag surgeries. Patient inclusion criteria comprised diagnosis of GO with disfiguring exophthalmos, Hertel exophthalmometry measurements stable for more than 6 months, patients in euthyroid status for more than 3 months, and a clinical activity score of <3. The exclusion criteria were as follows: prior orbital radiotherapy or prior orbital surgery; a medical history of eyelid, orbital, or intraocular inflammation or malignant neoplasm; age <20 years; trauma; compressive optic neuropathy; and pregnancy.

### 2.2. Orbital Fibroblast Isolation, Culture, and Treatment

Orbital fibroblasts were cultivated as previously reported [13]. Orbital tissues were minced and placed in plastic culture dishes with Dulbecco’s modified Eagle’s medium (DMEM) supplemented with 20% fetal bovine serum (FBS) and 50 IU/mL penicillin-streptomycin (Gibco^®^; Thermo Fisher Scientific, Waltham, MA, USA), allowing orbital fibroblasts to grow out. Monolayers were covered with DMEM supplemented with 10% FBS and serially passaged with gentle trypsin/ethylenediaminetetraacetic acid (EDTA) (Gibco^®^; Thermo Fisher Scientific, Waltham, MA, USA) treatment. These were incubated in a humidified incubator at 37 °C and 5% carbon dioxide (CO_2_).

The cells were passed every 3 days by trypsinization. The orbital fibroblasts were seeded into 6-well plates (Corning, Lowell, MA, USA). All experiments were performed with fibroblasts between the third and eighth passages from culture initiation. Three independent strains from different donors were used for the repeated experiments. Potential mesenchymal stem cells from the orbital fat tissue were isolated according to an isolation method for classical adipose-derived stem cells [14].

After treatment with IL-17A (up to 200 ng/mL), Z-YVAD-fmk (up to 10 lg/mL), or small interfering RNA (siRNA), the orbital fibroblasts were tested for viability using LIVE/DEAD Fixable Near-IR Dead Cell Stain Kit (Invitrogen, Carlsbad, CA, USA) and flow cytometry. The cell viability was above 85% and did not decrease following these treatments.

### 2.3. Immunohistochemical and Immunofluorescence Staining

The specimens collected from the GO patients and healthy controls were fixed in 4% buffered formalin and embedded in paraffin. Tissue cross-sections were prepared at a 6 μm thickness for immunohistochemical staining. After blocking the endogenous peroxidase activity in 3% H_2_O_2_, sections were incubated overnight with anti-NLRP3 mAb (Cryo-2, 1:200 dilution, adipoGen, Fuellinsdorf, Switzerland). The sections were washed with phosphate-buffered saline (PBS) and incubated with biotinylated secondary antibodies for 20 min. After washing with PBS, the sections were incubated with horseradish peroxidase-conjugated streptavidin for 20 min and processed with a DAB Substrate Kit (SK-4100, Vector Laboratories, Burlingame, CA, USA). Finally, they were counterstained with hematoxylin.

The orbital fibroblasts were cultured on six-well plates containing glass coverslips, and the cells were fixed and blocked for the immunofluorescence assay. The coverslips were incubated with anti-NLRP3 antibody (ab4207, 1:200 dilution, Abcam, Boston, MA, USA) overnight at 4° C. After three washes, the coverslips were processed with secondary antibodies conjugated to Alexa Fluor 488 (A11034, Invitrogen, Carlsbad, CA, USA). The nuclei were counterstained using DAPI (H-1500, Vector Laboratories, Burlingame, CA, USA). The glass slides were observed using a confocal microscope (LSM 800, Carl Zeiss, Oberkochen, Germany), and representative cells were imaged and analyzed. The staining intensity quantification was performed using ImageJ software.

### 2.4. Quantitative Real-Time Reverse Transcription PCR

Total RNA was isolated from orbital fat specimens using the iScript™ cDNA Synthesis Kit (Bio-rad, Inc., Hercules, CA, USA) following the manufacturer’s protocol. Complementary DNA was synthesized using 1 μg of total RNA incubated with random hexamers, 4 μL of 5x iScript Reaction Mix with 1 μL of iScript Reverse Transcriptase, and appropriate nuclease-free water to complete a total volume of 20 μL. The conditions used to obtain the cDNA were 25 °C for 10 min, 46 °C for 20 min, and 95 °C for 1 min. The cDNA was used as a template in quantitative real-time reverse transcription PCR (qRT-PCR) by the StepOne Real-Time PCR System (Thermo Fisher, Waltham, MA, USA). Published primers (Table 1) were used to amplify pro-caspase-1, NLPR3, and pro-IL-1β. The primers for glyceraldehyde 3-phosphate dehydrogenase (GAPDH) were obtained from Sino Biological, Inc. (Cat no. HP100003; Wayne, PA, USA). The mRNA levels of each target gene were normalized to GAPDH levels and provided as the fold induction. For each mRNA assayed, a sequence-specific standard curve was generated using 10-fold serial dilutions of standard PCR products. After standardizing the protocol for each gene, 20 μL PCR reactions were set up with final concentrations of 5 Mm of MgCl_2_, 5 μL of SYBR green mastermix, 1 μL of 1:10 dilute cDNA, and 1 μL (10 μΜ) of both forward and reverse primers. The reactions were cycled as follows: denaturation for one cycle at 95 °C for 10 min, 45 cycles of 95 °C for 15 s, 50 °C for 30 s, and 72 °C for 15 s, with fluorescence reading at 72 °C. The StepOne software generated a standard curve enabling identification of the gene copy numbers in each test sample. All PCRs were performed in triplicate.

### 2.5. Enzyme-Linked Immunosorbent Assay

IL-1β protein levels were measured using enzyme-linked immunosorbent assay (ELISA; R&D Systems, Minneapolis, MN, USA), following the manufacturer’s protocol. The assay was based on the competition between free cytokine and a cytokine tracer (cytokine linked to an acetylcholinesterase) for a limited number of cytokine-specific rabbit antiserum binding sites. The cytokine tracer and rabbit cytokine antiserum were added to the sample and incubated in the assay plate at 4 °C for 18 h. The wells were rinsed five times with washing buffer to remove any unbound reagents. Ellman’s reagent was added, and the color intensity in each well was measured using an ELISA Reader (FLUOstar Omega, BMG LABTECH, Ortenberg, Germany) at a wavelength of 450 nm. Samples were assayed in triplicate using a standard curve, as suggested by the manufacturer.

### 2.6. Western Blotting

Orbital fat samples were homogenized in RIPA lysis buffer (Merck KGaA, Darmstadt, Germany) with protease inhibitors. One hundred micrograms of total protein was electrophoresed on 10% denaturing polyacrylamide stacking gels and transferred to nitrocellulose membranes, which were blocked for 1 h at room temperature in 5% Blotto (PBS/0.1% Tween 20, and 5% milk). Mouse or rabbit (1 μg/mL) primary antibodies (proteins for caspase-1, NLPR3, and pro-IL-1β) (AdipoGen, Switzerland) were added in 5% Blotto overnight at 4 °C, washed with PBS/Tween 20, and the secondary antibodies, goat anti-rabbit IgG-horseradish peroxidase, rabbit anti-goat IgG-horseradish peroxidase, and goat anti-mouse IgG-horseradish peroxidase, were added for 1 h in 5% Blotto (AdipoGen, Switzerland). Membranes were washed in PBS/Tween 20. Detection was conducted using the enhanced chemiluminescence system.

### 2.7. IL-17A-Stimulated IL-1β Production from Orbital Fibroblasts via NLRP3 Inflammasomes

Orbital fibroblasts were grown to confluence in 6-well plates in Media 199 with 10% FBS. Upon reaching confluence, cultures were treated with IL-17A in different concentrations (0 ng/mL, 1 ng/mL, 10 ng/mL, and 100 ng/mL) for 0 h, 12 h, 24 h, and 48 h. The mRNA expressions of pro-IL-1β, caspase-1, and NLRP3 were measured using qRT-PCR. Mature IL-1β secretion into the culture supernatant was also evaluated using the ELISA assay previously described. The cultured cells were lysed, and the whole-cell extracts were assayed via Western blotting using antibodies against NLRP3 and the active form of caspase-1, following the protocol previously described.

Additionally, the cultures were pre-treated with caspase-1 inhibitor (Z-YVAD-fmk 1 and 10 μg/mL) for 30 min, and stimulated with IL-17A for 24 h. Again, IL-1β secretion into the culture supernatant was assayed using ELISA.

### 2.8. Validation of NLRP3 Inflammasome Function Using RNA Interference for NLRP3 Expression Knockdown

To reduce endogenous NLRP3 expression, orbital fibroblasts were transfected with small interfering RNA (siRNA) oligonucleotides using Lipofectamine 2000 reagent (Thermo Fisher, USA). siNLRP3 plasmids (PLKO-AS2-puro-shNLRP3#1) were kindly provided by Dr. Hsu’s laboratory in Taiwan. The cells were transfected with either a specific siRNA oligonucleotide against NLRP3 (siNLRP3) or a nonspecific control siRNA oligonucleotide (siControl). After incubation, the fibroblasts were treated with IL-17A (100 ng/mL) for 24 h; the cell culture medium was collected for pro-IL-1β ELISA analysis, and mRNA and protein of NLRP3 levels were analyzed using qRT-PCR and Western blotting.

### 2.9. Statistical Analysis

At least three cell strains from different individuals were used in all experiments, and all sample assays were carried out in triplicate. The experimental results are shown as the mean ± SD calculated from normalized measurements. Analysis of variance or the Student’s t-test were used to determine statistical significance (*p* < 0.05) using the software SPSS version 26.0 (SPSS Inc, Chicago, IL, USA).

## 3. Results

### 3.1. NLRP3 Inflammasomes Are Prevalent in GO Orbital Fibroblasts

**Figure 1:**
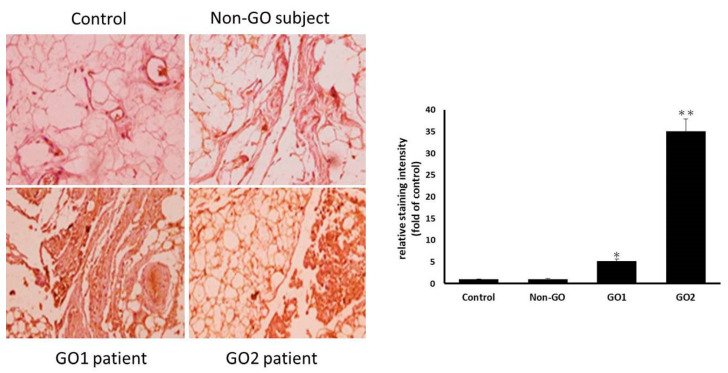
Representative images of immunohistochemical staining for NLRP3 in orbital connective tissue from a non-GO subject and 2 GO patients, using IgG as negative control, showing that more obvious NLRP3 expression (brown staining) was noted in GO orbital tissues. Advanced quantitative analysis revealed that NLRP3 expression was higher in the orbital connective tissue from GO patients than in that from control subjects. The bar charts show mean data of relative staining intensity of NLRP3 presented as the fold of control. Data are presented as mean ± SD from at least three independent experiments. * *p* < 0.05, ** *p* < 0.01.

**Figure 2:**
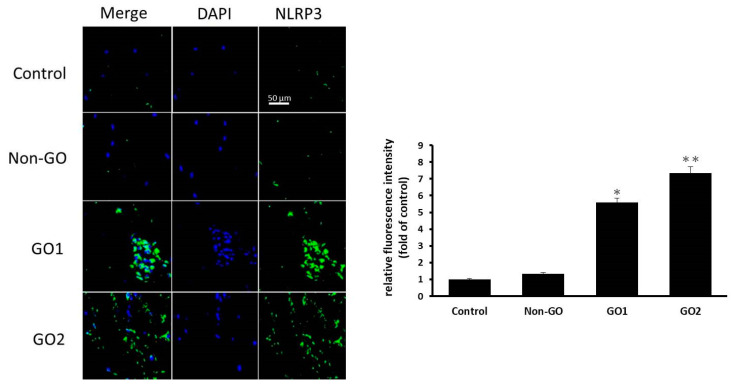
Representative images of immunofluorescence staining for NLRP3 in cultured orbital fibroblasts from a non-GO subject and 2 GO patients, using IgG as negative control, showing higher NLRP3 expression (green staining) in GO orbital tissues. Advanced quantitative analysis revealed that NLRP3 expression was higher in the orbital connective tissue from GO patients than in that from control subjects. The bar charts show mean data of relative fluorescence intensity of NLRP3 presented as the fold of control. Data are presented as mean ± SD from at least three independent experiments. * *p* < 0.05, ** *p* < 0.01.

In the NLRP3 immunohistochemical assay using IgG as a negative control, the connective tissue in the orbital tissues of patients with GO was observed to exhibit obvious NLRP3 inflammation compared with that of the healthy non-GO subject group (Figure 1). The cell culture immunofluorescence staining displayed in Figure 2 also showed that fibroblasts in GO orbital connective tissue exhibited higher NLRP3 inflammasome expression than those in orbital connective tissue from the non-GO group.

These results demonstrate that NLRP3 inflammasomes are prevalent in the orbital connective tissue and cultured fibroblasts from patients with GO, suggesting that orbital fibroblasts in patients with GO may be directly influenced by IL-17A, and that a further inflammatory response is induced through NLRP3 inflammasomes to promote the formation of GO.

### 3.2. IL-17A Promotes Orbital Fibroblasts to Secrete IL-1β

**Figure 3:**
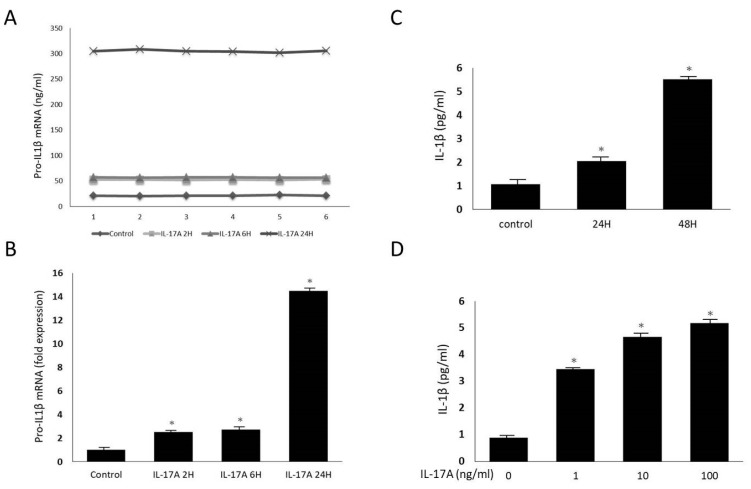
Time- and dose-dependent effects of IL-17A on the production of pro-IL-1β mRNA and IL-1β in GO orbital fibroblasts. (**A**,**B**) GO orbital fibroblasts were treated with 100 ng/mL IL-17A for 0 h, 2 h, 6 h, or 24 h. Pro-IL-1β mRNA expression was determined by real-time PCR. The samples number was 6 and 3 independent experiments were implemented. The expression of pro-IL-1β mRNA secreted by GO orbital fibroblasts gradually increased with the increase in IL-17A stimulation time. (**C**) GO orbital fibroblasts were treated with 100 ng/mL IL-17A for 0 h, 24 h, or 48 h. IL-1β expression was determined by ELISA. (**D**) GO orbital fibroblasts were treated with different doses (0 ng/mL, 1 ng/mL, 10 ng/mL, and 100 ng/mL) of IL-17A for 24 h. The protein levels of IL-1β in the culture supernatant were measured by ELISA. It was observed that with the increase in the duration of IL-17A stimulation, GO orbital fibroblasts secreted more IL-1β, and the higher the concentration of IL-17A used for stimulation, the more IL-1β was produced. Data are presented as mean ± SD from at least three independent experiments. * *p* < 0.05.

To evaluate the proinflammatory effect of IL-17A on orbital fibroblasts, primary fibroblasts derived from the GO orbital tissues were treated with different concentrations of IL-17A for different time periods. Many proinflammatory molecules play important roles in the pathogenesis of GO, including the proinflammatory cytokine, IL-1β [15]. We hypothesized that IL-17A might be an inducer of inflammatory mediators in orbital connective tissue. To verify this hypothesis, orbital fibroblasts were first incubated with IL-17A (100 ng/mL) for 24 h. The expression of pro-IL-1β mRNA was measured using RT-PCR and IL-1β protein production was detected by ELISA. Six subjects were randomly chosen from each group. By increasing the duration of IL-17A stimulation, the expression of pro-IL-1β mRNA secreted by GO orbital fibroblasts increased in a time-dependent manner (Figure 3A,B). Consistent with these results, the ELISA revealed that concentrations of IL-1β in the orbital fibroblast culture supernatant were significantly increased after IL-17A stimulation in a dose- and time-dependent manner (Figure 3C,D). These results revealed that IL-17A induces pro-IL-1β transcription and IL-1β production in orbital fibroblasts from patients with GO. It might indicate that IL-17A could promote proinflammatory cytokine production and trigger a future inflammatory process in the pathogenesis of GO.

### 3.3. IL-17A Activates NLRP3 and Caspase-1 Production in GO Orbital Fibroblasts

**Figure 4:**
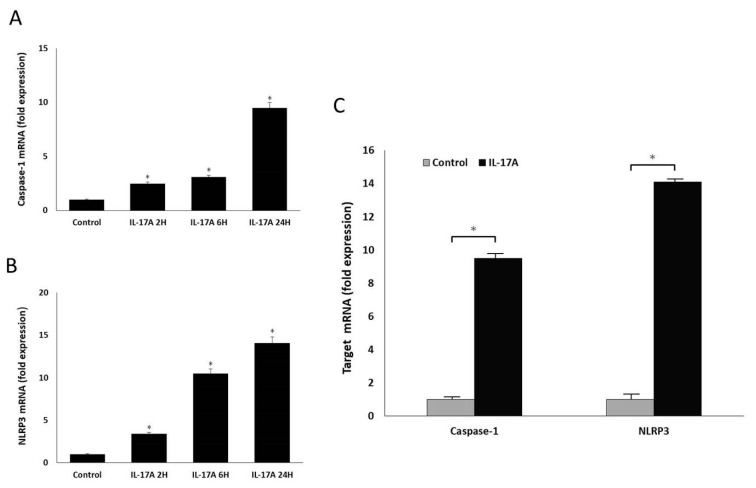
Time-dependent effects of IL-17A on the production of caspase-1 and NLRP3 in GO orbital fibroblasts. (**A**,**B**) GO orbital fibroblasts were treated with 100 ng/mL IL-17A for 0 h, 2 h, 6 h, and 24 h. Caspase-1 and NLRP3 mRNA expression were detected by real-time PCR. (**C**) GO orbital fibroblasts were stimulated with 100 ng/mL IL-17A for 24 h, and the mRNA expressions of caspase-1 and NLRP3 were detected by real-time PCR, revealing that IL-17A can indeed promote orbital fibroblasts to increase caspase-1 and NLRP3 mRNA expression. Data are presented as mean ± SD of at least three independent experiments. * *p* < 0.05.

**Figure 5:**
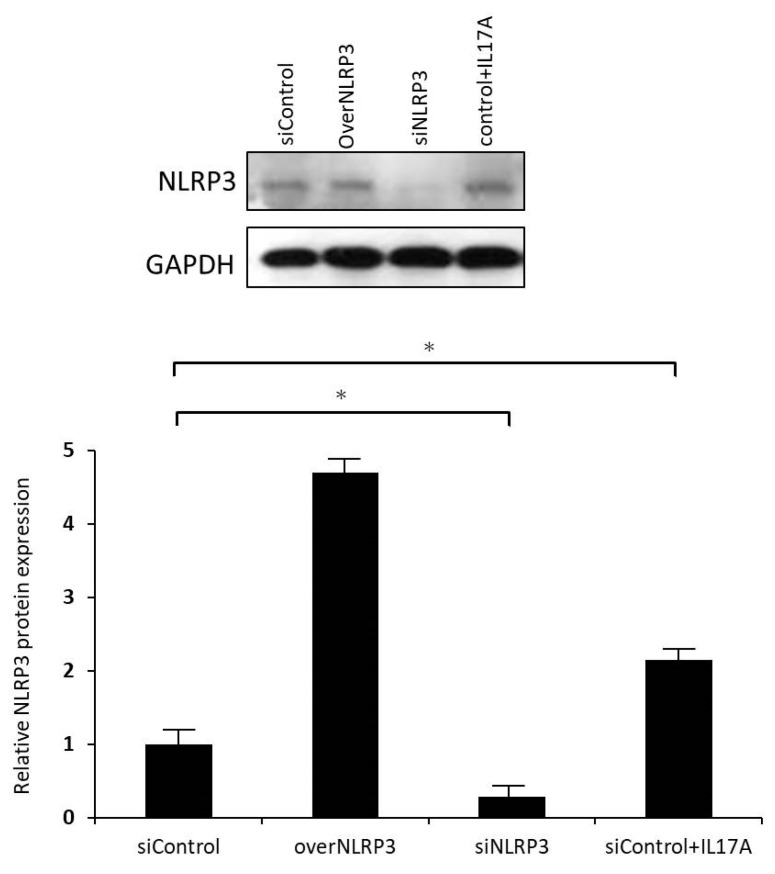
IL-17A enhances NLRP3 protein expression in GO orbital fibroblast. GO orbital fibroblasts were treated with or without 100 ng/mL IL-17A for 24 h. NLRP3 protein production was assessed by Western blot analysis. siControl means nonspecific control siRNA oligonucleotide. siNLRP3 represents specific siRNA oligonucleotide against NLRP3. OverNLRP3 means GO orbital fibroblasts were transfected with NLRP3-expressing plasmid and induce overexpression of NLPR3. Data are presented as mean ± SD of at least three independent experiments. * *p* < 0.05.

Activation of the NLRP3 inflammasome would promote the maturation of pro-IL-1β and subsequent IL-1β secretion. Next, we studied how NLRP3 interacts with IL-17A to trigger IL-1β production and to investigate the possible mechanism of NLRP3 overexpression in the orbital tissue from patients with GO. We measured the synthesis of NLRP3 and caspase-1 in GO orbital fibroblasts stimulated by IL-17A. The results demonstrated that the orbital fibroblasts were able to upregulate mRNA expressions of caspase-1 and NLRP3 upon IL-17A stimulation (Figure 4). Similarly, IL-17A could also induce intensified protein expressions of activated caspase-1 and NLRP3 (Figure 5), implying that activation of NLRP3 inflammasomes is closely related to IL-17A. Since IL-17A is a major proinflammatory cytokine in the orbital tissue from patients with GO [4], our in vitro data indicated that the overexpression of NLRP3 could be stimulated by IL-17A in GO orbital tissue.

### 3.4. Z-YVAD-Fmk Inhibited IL-17A Induced IL-1β Production in GO Orbital Fibroblasts

**Figure 6:**
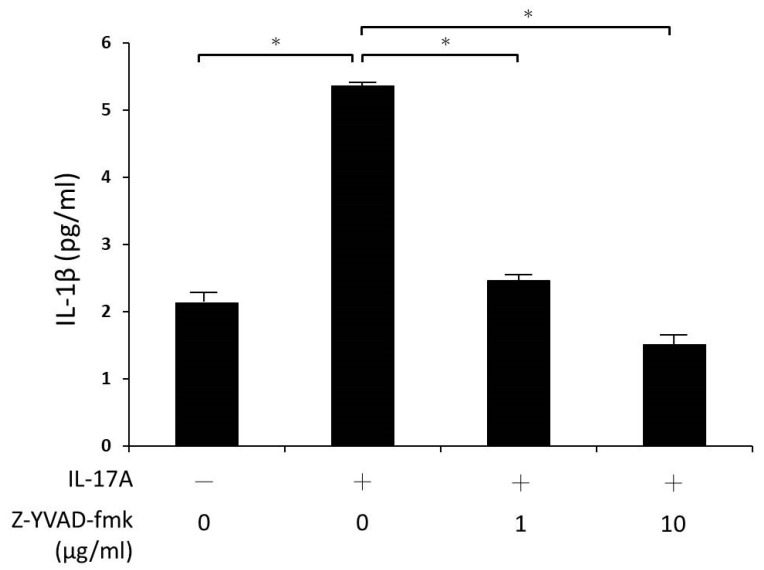
Effects of caspase-1 inhibitor Z-YVAD-fmk on IL-17A induced IL-1β production in GO orbital fibroblasts. GO orbital fibroblasts were stimulated with 100 ng/mL IL-17A for 24 h with or without a 30 min pretreatment with Z-YVAD-fmk (1 or 10 μg/mL). IL-1β expression was examined by ELISA. Data are presented as mean ± SD of at least three independent experiments. * *p* < 0.05.

Furthermore, we used Z-YVAD-fmk, a specific inhibitor of caspase-1, to confirm the role of caspase-1 in IL-17A-induced IL-1β production. Orbital fibroblasts were first treated with 1 μg/mL or 10 μg/mL Z-YVAD-fmk for 30 min and then stimulated with IL-17A for 24 h. The supernatant was analyzed via ELISA. It was observed that IL-1β secretion was effectively suppressed by Z-YVAD-fmk after IL-17A stimulation in a concentration-dependent manner. When the Z-YVAD-fmk concentration was increased from 0 to 10 μg/mL, the decreased secretion of IL-1β also increased as its concentration increased (Figure 6). These results indicated that inhibition of IL-1β expression could be one of the mechanisms for Z-YVAD-fmk to prevent the inflammatory process in GO.

### 3.5. siNLRP3 Inhibited IL-17A-Induced IL-1β Production in GO Orbital Fibroblasts

**Figure 7:**
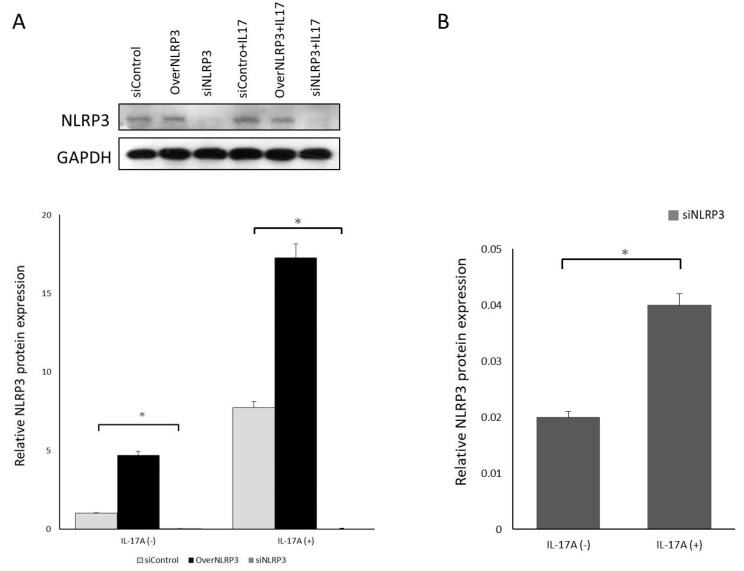
NLRP3 overexpression or knockdown. (**A**) GO orbital fibroblasts were treated with or without 100 ng/mL IL-17A for 24 h. NLRP3 protein production was assessed by Western blot analysis. It revealed that siRNA transfection effectively downregulated the expression of NLRP3 protein with or without IL-17A stimulation. (**B**) Relative NLRP3 protein expression between IL-17A unstimulated and IL-17A stimulated samples when siNLRP3 was applied. The significant result implied that IL-17A plays an essential role in regulating NLRP3 expression. siControl means nonspecific control siRNA oligonucleotide. NLRP3-expressing plasmid and induce overexpression of NLPR3. Data are presented as mean ± SD of at least three independent experiments. * *p* < 0.05.

**Figure 8:**
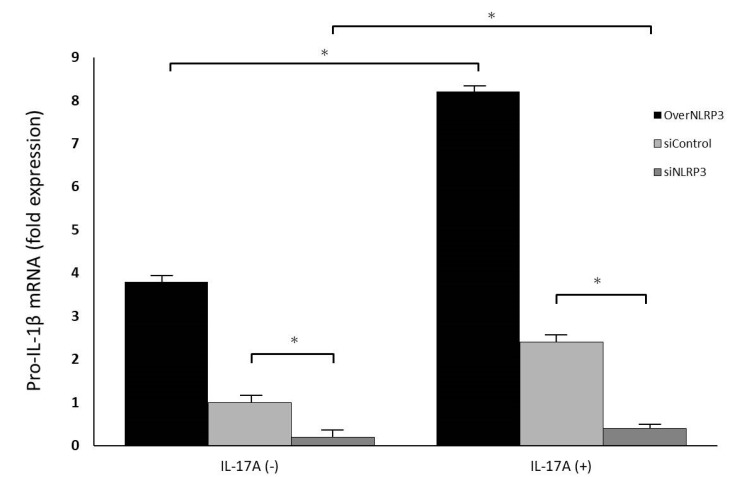
IL-17A induced pro-IL-1β expression in GO orbital fibroblasts with NLRP3 overexpression or knockdown. GO orbital fibroblasts were treated with or without 100 ng/mL IL-17A for 24 h under NLRP3 overexpression or knockdown. Pro-IL1β mRNA expression was detected by real-time PCR. The result also showed that siNLRP3 significantly inhibited the expression of pro-IL1β mRNA regulated by IL-17A. Data are presented as mean ± SD of at least three independent experiments. * *p* < 0.05.

To further understand the role of NLRP3 in IL-17A in promoting orbital fibroblasts to secrete IL-1β, we used siRNA for NLRP3 inflammasome knockdown. Regardless of the presence or absence of IL-17A stimulation, the transfection of siRNA effectively downregulated the expression of NLRP3 mRNA and protein (Figure 7). Additionally, NLRP3 inflammasome knockdown significantly inhibited the expression of pro-IL-1β mRNA regulated by IL-17A (Figure 8), indicating that the NLRP3 inflammasome might play a critical role in IL-17A stimulation in orbital fibroblasts. In this experimental part, we speculated that IL-17A would increase NLRP3 and caspase-1 expression in GO orbital fibroblasts, with caspase-1 further inducing IL-1β production. Inhibition of NLRP3 or caspase-1 can decrease IL-1β secretion.

## 4. Discussion

The manifestation of GO is an autoimmune and chronic inflammatory process of the orbital tissues that can result in disfigurement and potential visual loss. Inflammatory cytokines play an important role in the pathogenesis of GO. IL-1β is a potent proinflammatory cytokine that is produced by fibroblasts and macrophages, promoting hyaluronic acid synthesis, prostaglandin E2 accumulation, and cytokine secretion (including IL-6, IL-8, and MCP-1) to induce adipogenesis. Accumulation of hyaluronic acid leads to the formation of GO [5]. IL-1β can promote an inflammatory response by regulating gene expression, which in turn attracts and activates other cells of the acquired immune system. Th17 cells also have IL-1β receptors, which release IL-17A when they are stimulated. IL-1β also functions in synergy with IL-23 to promote the production of IL-17A and related cytokines from Th17 cells. Therefore, when excessive IL-1β production is continuously stimulated, it will strengthen the Th17-based immune response [12]. Our results revealed that orbital fibroblasts are an important source of IL-1β, especially in the orbital tissue of patients with GO; similarly, previous AMD studies also confirmed that IL-17A can indeed promote RPE cells to induce IL-1β secretion in inflammatory responses [12].

Th17 cells are transformed from undifferentiated CD4+ T cells. When the nucleus contains lineage-specific transcription factor orphan nuclear receptor (retinoic acid receptor-related orphan receptor γt, RORγt) after receiving external stimuli, cytokines are secreted to induce this undifferentiated T cell to further transform into Th17 cells capable of secreting IL-17A to generate a protective immunity response [16,17,18,19]. Recent studies have shown that there is a significantly increased number of Th17 cells, a higher concentration of IL-17A, and a larger number of IL-17A receptors in the orbital tissue of patients with GO, and relative amounts of inflammatory cytokines, such as IL-6 and IL-8, are also increased, promoting the accumulation of ECM in orbital fibroblasts [4]. Additionally, IL-17A can also cooperate with other cytokines, such as IL-23, IL-6, IL-1β, and TGF-β, to regulate the acquired immune system and inflammatory response in the connective tissue of the orbit, resulting in orbital tissue intensification and remodeling [4]. Our study also showed that IL-17A can promote orbital fibroblasts to secrete IL-1β, and may induce further GO development.

An inflammasome is a complex that comprises multiple proteins in the cell, which is the reaction platform necessary for caspase activation. Intracellular infection or stimulation can activate the inflammasome and induce the maturation and secretion of inflammatory cytokines, such as IL-1β, prompting a series of innate immune responses [20]. The NLRP3 inflammasome consists of three parts. The first part is NLRP3, which mainly controls the specificity and activity of the inflammasome. NLRP proteins contain LRR sequences that function like toll-like receptors on the cell membrane, and are therefore related to matrix identification. NLRP also contains an interaction region, enabling it to bind to the second part of the skeleton protein ASC (apoptosis-associated speck-like protein containing a CARD), and ASC to combine to the third part of pro-caspase-1. When the inflammasome is activated, pro-caspase-1 is cleaved to caspase-1, and then, pro-IL-1β is cleaved to become active IL-1β [21].

Presently, the detailed mechanism of NLRP3 inflammasome activation is unclear, but may occur as follows: Firstly, through ROS, stimulants can promote the production of active oxides by NADPH oxidase in phagosomes [22]. Secondly, when cells are damaged and activated, ATP is released to the outside of the cell, a process that stimulates immune cells. ATP binds to the P2X7 receptor, leading to the activation of phagocytic cells and monocyte-producing NLRP3 inflammasomes, promoting IL-1β production. Part of the inflammasome activation occurs through pannexin-1, and it also causes outflow of intracellular potassium ions. Inhibiting potassium ion outflow can reduce the production of IL-1β [22,23]. Additionally, when the extracellular calcium-sensing receptor senses extracellular calcium ions, it can regulate the increase in the intracellular calcium ion concentration, or decrease the intracellular cyclic AMP concentration, to activate the NLRP3 inflammasome. Protein kinase R has been shown to be involved in inflammasome activation, and is an interferon-induced protein that regulates high-mobility group box (HMGB) protein release [24]. After inflammasome activation, caspase-1 can further induce other cytokine precursors (including IL-1β) and promote further inflammatory reactions, which may even lead to the formation of GO. Research findings also indicate that the ROS concentration is higher in GO orbital fibroblasts, and that orbital fibroblasts are more sensitive to changes in oxidative stress, all of which play an important role in the pathogenesis of GO [10,11]. Our experimental results showed that IL-17A could promote orbital fibroblasts to induce NLRP3 inflammasomes and caspase-1 activation, thereby increasing IL-1β secretion. Therefore, we supposed that in the pathogenesis of GO, when Th17 cells are stimulated by ROS, IL-17A is released to activate the orbital fibroblasts, by which NLRP3 inflammasomes are activated, with caspase-1 further promoting fibroblasts to secrete IL-1β to form a subsequent immune inflammatory response.

Caspase-1 can convert immature pro-IL-1β precursors to mature IL-1β, whereas caspase-1 cleaves and activates pro-caspase-1 through the NLRP3 inflammasome complex. Although the activation of caspase-1 and IL-1β dominated by NLRP3 inflammasomes in RPE cells has been demonstrated in previous experiments [25], there is no relevant experiment in the study of GO to explain how NLRP3 promotes IL-1β secretion in orbital fibroblasts. Based on our results, it is known to us that when NLRP3 receives IL-17A stimulation and activation, pro-caspase-1 will further transform into caspase-1, which will then activate pro-IL-1β to release IL-1β; subsequent IL-1β-induced adipogenesis, glycosaminoglycan accumulation, and secretion of IL-6 and IL-8 cytokines may lead to the formation of GO. At the same time, in our experiments, the caspase-1 inhibitor Z-YVAD-fmk could effectively inhibit the activity of caspase-1 and reduce the production of pro-IL-1β mRNA. It was also noticed that IL-17A-induced orbital fibroblast IL-1β secretion is regulated by caspase-1.

The current treatment of GO mainly includes high-dose corticosteroid injection orbital radiation therapy, and orbit decompression surgery. Considering the gradual understanding of the pathogenesis of GO, including the development of the orbital inflammation response pathway caused by global autoimmunity, optimized medical treatments should inhibit this autoimmune response. For example, infliximab and adalimumab are TNF-specific monoclonal antibodies [26,27]; etanercept (a TNF receptor-IgG fusion molecule) [28] and pentoxifylline [29] are both aimed at inhibiting TNF-α. Tocilizumab targets IL-6, and lerdelimumab suppresses TGF-β. Additionally, rituximab acts on B cells, and is currently used clinically to treat thyroid exophthalmos [30,31].

However, high-dose corticosteroids have limited therapeutic effects, and are associated with safety concerns and several side effects, such as iatrogenic Cushing’s disease, diabetes, osteoporosis, hypertension, peptic ulcer, and infections [32]. Presently, the results of large-scale clinical experiments have shown that type I IGF-1R inhibitors are also effective treatment strategies. Teprotumumab is a human IGF-1R monoclonal antibody inhibitor given intravenously every 3 weeks for patients with moderate to severe acute GO. After six consecutive months of treatment, symptoms rapidly improve. The only side effect is that patients with diabetes may be at risk for hyperglycemia; however, this can be medically controlled [33]. According to our study, IL-17A and NLRP3 significantly influence the pathogenesis of GO. In the future, developing antagonists against IL-17A or IL-17A receptors, manufacturing IL-1β monoclonal antibodies, and conducting NLRP3 inhibitor or caspase-1 inhibitor-related immunotherapy studies aimed at finding a way to block the pathway of IL-17A promoting IL-1β secretion, may help prevent the development of GO. It is believed that these advances may prevent the formation of GO in the future.

A limitation of the present study is the fact that GO orbital adipose tissue specimens were used as the experimental basis for understanding the relationship between IL-17A and NLRP3 inflammasomes and the correlation between subsequent inflammatory cytokines IL-1β and autoimmune eye diseases. However, there is a lack of serological studies on Th17, IL-17A, NLRP3, and IL-1β. Therefore, it cannot be confirmed whether the results of orbital GO fibroblast cultures would be consistent with serology results currently. Additionally, a valid in vivo comparison and evaluation of differences in IL-17A, caspase-1, and IL-1β levels in the orbital tissues of GO and control groups is lacking. Thirdly, the GO orbital fibroblasts were obtained from patients with inactive GO because of the surgical indication. Further studies must be conducted using specimens from patients with active GO to compare the results between different disease stages. These experimental limitations should prompt further research aiming to gain a more detailed understanding.

## 5. Conclusions

Our study provided preliminary evidence that IL-17A and NLRP3 inflammasomes may play an essential role in the GO pathogenesis. It also illustrated that IL-17A could promote mature IL-1β production from orbital fibroblasts through NLRP3 inflammasome activation in GO. The IL-1β-induced cytokines promoted inflammation and autoimmunity to cause GO formation. Additionally, the present experiment may contribute to our understanding of orbital immunity and GD, providing us with relevant targets in the optimization of the treatment of GO.

## Figures and Tables

**Table 1:** Primer sequences used for real-time PCR analyses.

Target	Forward	Reverse
GAPDH	5′-TTCGACAGTCAGCCGCATCTTCTT-3′	5′-GCCCAATACGACCAAATCCGTTGA-3′
IL-1β	5′-CGAATCTCCGACCACCACTAC-3′	5′-GCACATAAGCCTCGTTATCCC-3′
NLRP3	5′-ACTCTGTGAGGGACTCTTGC-3′	5′-GGTCGCCCAGGTCATTGTT-3′
Pro-caspase-1	5′-TGAAGGACAAACCGAAGG-3′	5′-GAAGAGCAGAAAGCGATA-3′
Pro-IL-1β	5′-AGCTACGAATCTCCGACCAC-3′	5′-CGTTATCCCATGTGTCGAAGAA-3′

## Data Availability

Data available on request due to restrictions privacy.
